# Nerve Transfer for Restoration of Ulnar Fingers Flexion Through Pronator Teres Motor Branch: A Cadaveric Feasibility Study

**DOI:** 10.1016/j.jhsg.2025.100844

**Published:** 2025-11-01

**Authors:** Paolo Titolo, Alessandro Crosio, Andrea Fanecco, Erica Cavalli, Francesca Vincitorio, Julien Teodori, Giulia Colzani, Nathalie Bini, Mario Ronga, Michele Rosario Colonna, Bruno Battiston, Davide Ciclamini

**Affiliations:** ∗ Department of Orthopaedics and Traumatology, Hand and Microsurgery Unit, Orthopaedic Trauma Center CTO - A.O.U. Città della Salute e della Scienza, Turin, Italy; † Department of Plastic Surgery and Hand Surgery, IRCCS San Gerardo dei Tintori, Monza, Italy; ‡ Department of Neurosciences and Mental Health, A.O.U. Città della Salute e della Scienza, Turin, Italy; § Department of Pediatric Orthopeadic Surgery, A.O.U. Città della Salute e della Scienza, Turin, Italy; ‖ Department of Health Sciences, University of Eastern Piedmont, Novara, Italy; ¶ Department of Human Pathology of the Adult, the Child and the Adolescent, University of Messina, Messina, Italy

**Keywords:** Brachial plexus injury, Nerve transfer, Peripheral nerve injury, Tetraplegia, Ulnar nerve

## Abstract

**Purpose:**

Injury to the ulnar nerve above the elbow or other conditions such as brachial plexus injury or cervical spinal cord injury can also affect the extrinsic finger flexors. Numerous techniques have been described to treat this problem, but no specific nerve transfer has been proposed.

**Methods:**

Here, we describe the transfer of the motor branch of the pronator teres (PT) to reinnervate flexor digitorum profundi of the ulnar nerve. Eleven fresh frozen upper limbs were used. The anatomical characteristics of the branches to the flexor digitorum profundus and PT were recorded.

**Results:**

In all cases, the superficial branch of the PT resulted as the longest between the two and, therefore, was used as a donor. It was passed under the flexor pronator muscles to reach the ulnar nerve. The recipient motor branch was dissected proximally, and a direct nerve suture was made between the two stumps.

**Conclusions:**

In all specimens, a direct tension free neurorrhaphy was possible, located close to the target muscle.

**Clinical relevance:**

This specific procedure can be suggested in cases where the hand is partially involved to allow patients to regain or strengthen fingers flexion. Direct clinical application should support and confirm this novel procedure.

Peripheral nerve injuries (PNIs) are frequent and troublesome conditions to deal with. Injuries afflicting the ulnar nerve (UN) are the most common form of upper-extremity PNIs and can cause remarkable loss of power grip, pinch strength, and hand dexterity.^1^^,^^2^

Gripping tasks, power grip (PG) especially, are indispensable for work and port but, mostly, for daily activities; performing PG, the object is wrapped by the hand in such a way that grip forces are exerted on its entire circumference by the palm, the fingers, and the thumb. The PG is usually performed when intense effort is needed, such as wielding heavy tools or handles.^3^ Much of the force exerted by the hand during PG is provided by the last two fingers, the musculature of which is mostly innervated by UN.^4^ In case of a segmental nerve loss, nerve reconstruction techniques, especially nerve autografts, are employed with excellent results; however, graft sustain to the regenerative process diminishes as the distance to cover increases; it is stated that clinical improvement decreases in grafts of over 5 cm.^5^

Despite improvements in microsurgical repair techniques, UN PNIs localized at the elbow are traditionally associated with poor prognosis.^6^ Valid alternatives are nerve transfer techniques, by which a redundant and expendable donor is killed to recover an essential motor function; because the chosen donor branch is closer to the target muscle, reinnervation time is shortened and the chances of recovery are increased.^7^ New nerve transfers techniques for UN PNIs have been developed over the years, improving patient outcomes.^1^ The motor branch of the UN to flexor digitorum profundus (FDP) supplies the third, fourth, and fifth digits and is a potential recipient nerve that has not yet received much attention in the literature. Indeed, in case of high median nerve (MN) PNIs, the UN allows complete flexion of the third, fourth, and fifth digits, resembling the posture of the “pointing finger”.^8^ The aim of this study is to describe a new nerve transfer technique using a motor branch of the MN to the pronator teres (PT) as a donor to recover finger flexion for the fourth and fifth digits in case of high UN injuries or incomplete brachial plexus injuries to improve grip strength. We report here our anatomical findings.

## Materials and Methods

**Figure 1:**
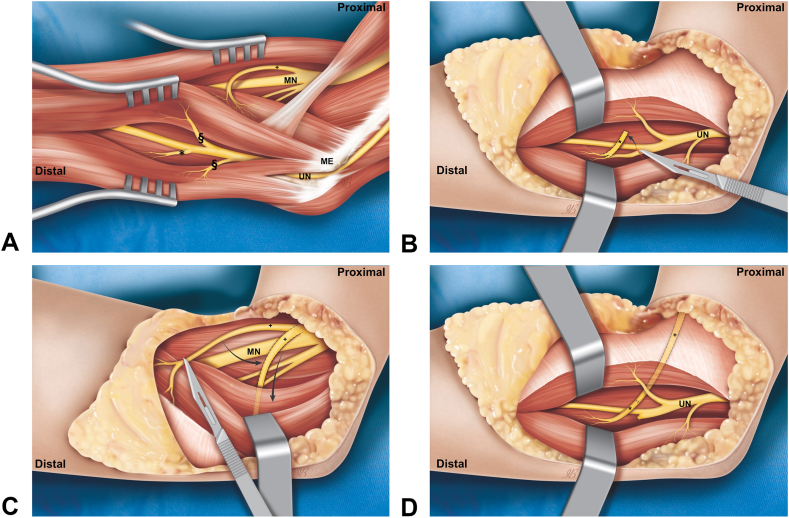
A schematic representation of the procedure. **A** Anatomical representation of branches for pronator teres from MN and branches of the UN for FCU (§) and flexor digitorum profundi (∗). **B** Superficial branch for pronator teres (+) is dissected and cut as distal as possible into muscle belly. **C** The UN motor branch to the flexor digitorum profundi is dissected and cut from its origin. **D** Nerve coaptation is performed in UN zone, the donor branch is passed underneath a sub muscular tunnel. ME, medial epicondyle; MN, median nerve; UN, ulnar nerve.

**Figure 2:**
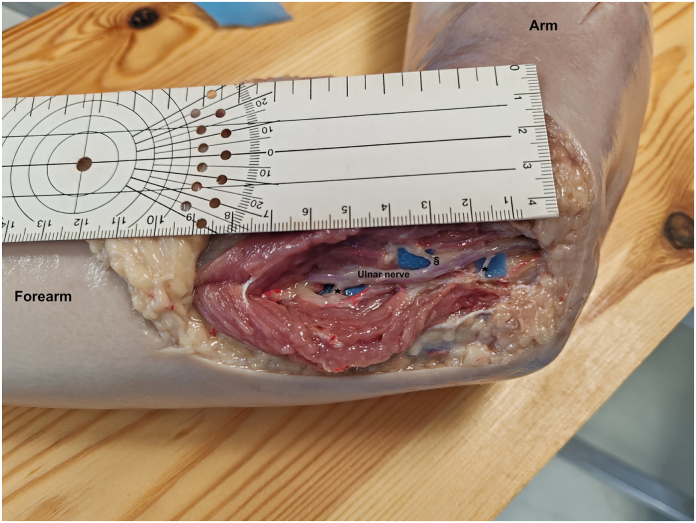
Dissected UN with outlined motor branches to the FCU (∗) and flexor digitorum profundi (§).

Eleven upper limbs, from eleven fresh frozen human cadavers, were dissected for the purpose of this study. No distinction of age, gender, or side was made. The dissections were performed under loupe magnification. All measurements were performed with the elbow in full extension. The UN and the ulnar motor branches to FDP (UMBsFDP) were dissected first (Fig. 1A). The UN was identified at the cubital tunnel, which was released, and followed distally until the branches to the flexor carpi ulnaris (FCU) and FDP were reached. A ruler was employed to measure the distance between the medial epicondyle and the origin of the UMBsFDP from the UN, the length of the UMBsFDP (from their origin from the UN to their entry into the muscle belly), and the diameter; we also noted from which side of the UN the branches emerged (ulnar, radial, or posterior) (Fig. 2).

**Figure 3:**
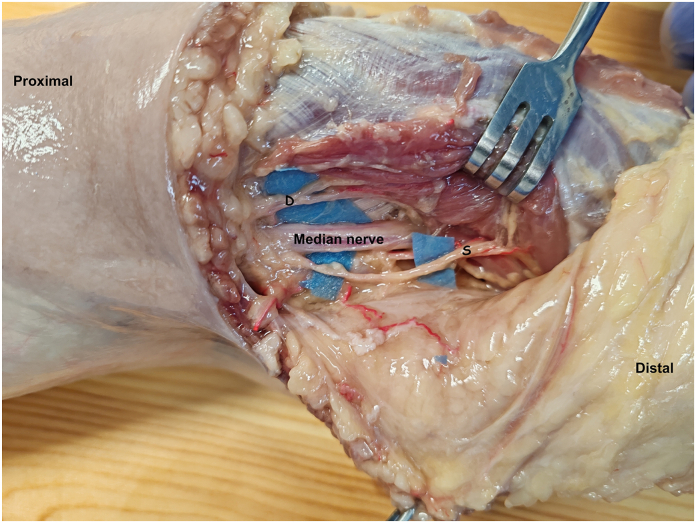
Dissected MN at the cubital fossa in which the two motor branches for both heads of pronator teres are highlighted. The superficial branch (S) appears to be longer than the deep branch (D).

The MN at the cubital fossa was then exposed and the origin of the motor branches to the pronator teres were identified (Fig. 1A). The number of motor branches and their site of origin from the MN were marked. For each branch, the distance between its origin from the MN and the medial epicondyle, its length from their origin from the MN and the muscle entry point, and the diameter were recorded (Fig. 3). The longest branch was chosen to perform the nerve transfer.

The dissection of the motor branch was continued into the muscle as distal as possible. Afterwards, a tunnel was created underneath the PT, and the donor nerve was passed through this (Fig. 1A, C).

**Figure 4:**
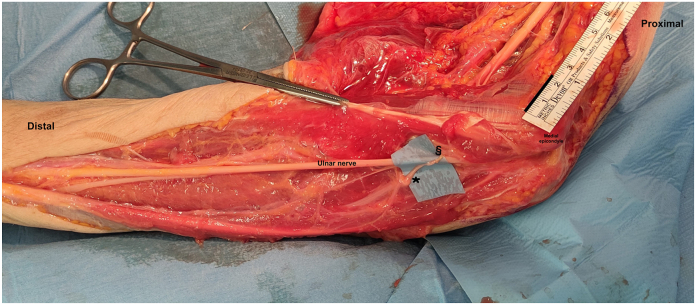
The site of nerve coaptation of pronator teres motor branch (§) close to the origin of motor branches for flexor digitorum profundi form UN (∗).

The most proximal branch to the FDP was chosen as recipient and dissected as proximal as possible till its origin from the UN, where it was sectioned (Fig. 1B). Once close, the receiving nerve end was trimmed and sutured in a tension free end to end manner with 8-0 non-absorbable suture as close as possible to muscle entry point (Fig. 1D). The nerve coaptation point was choses so that no traction was created on suture site during fingers extension that provoked distal translation of the nerve stump for FDPs. Consequently, the suture was performed with all long fingers extended as noted in the Figure 4. All measurements and procedures were photographed. The nerve transfer procedure is schematized in the Figure 1.

## Results

In all samples, the most proximal branch for FDP was identified and dissected. In two cases, the required motor branch originated from the trunk of the FCU. In the other nine samples, the most proximal motor branch for FDP was found exiting radially from the UN surface at a mean distance of 4.9 cm from medial epicondyle (min: 3, max: 7 cm). The mean diameter of all UMBsFDP was 0.2 cm, and their mean length was 3.8 cm (min: 2, max: 5 cm). The UMBsFDP originated from the radial aspect of the UN in all but three specimens that exited toward the ulnar side. Data are summarized in the Table 1.

**Table 1:** Morphological Characteristics of Motor Branches for FDP in Samples

Sample	No of Motor Branches	Distance From Medial Epicondyle to the Branch Origin (cm)	Branch Length (cm)	Diameter (cm)	Origin Position from the UN
1	1	4	2	0.2	Ulnar
2	1	3	2	0.2	Radial
3	1	1.3	5.5	0.2	Common with FCU
4	1	5.5	3.5	0.2	Common with FCU
5	1	7	4.5	0.3	Radial
6	1	5	3.5	0.2	Radial
7	1	6	4	0.2	Radial
8	1	5.5	5	0.2	Radial
9	1	6	4.5	0.3	Radial
10	1	3.5	4.5	0.3	Ulnar
11	1	4	4.5	0.3	Ulnar

Both superficial and deep heads of PT were observed during the dissection; the PT was innervated by two main motor branches arising from the MN in all specimens. The deep branch to the pronator teres originated at the level of intercondylar line (min: –3, max: 3 cm). Their mean diameter was 0.2 cm (min: 0.2, max: 0.4 cm), and their mean length was 4.2 cm (min: 2.5, max: 6 cm). The collected data regarding MBPT are summarized in the Table 2. Otherwise. the superficial branch originated more proximally (–2.6 cm proximally; min –2 cm, max –4 cm). The mean diameter was 0.3 (min 0.2, max 0.3 cm), and its mean length was 4.5 cm (min 3 cm, max 7 cm). As stated above, the longest motor branch of two was transferred. In all specimens, this was the superficial one. In all our dissections, both heads of PT were present; none of the specimens displayed any anomalous muscle or any abnormal nerve connection, such as the Marinacci or Martin-Gruber anastomosis.

**Table 2:** Morphological Characteristics of Pronator Teres Motor Branches

Sample	Innervation	Distance From Medial Epicondyle to the Branch Origin (cm)	Branch Length (cm)	Diameter (cm)
1	Deep	0	4	0.2
	Superficial	–4	5	0.3
2	Deep	0	5.5	0.2
	Superficial	–3.5	4	0.2
3	Deep	–0.2	2.5	0.3
	Superficial	–2.5	4	0.3
4	Deep	1	5.5	0.2
	Superficial	–2	3	0.3
5	Deep	–1	5	0.2
	Superficial	–4	7	0.2
6	Deep	–1.5	2.5	0.2
	Superficial	–3.8	4	0.2
7	Deep	3	3,5	0.2
	Superficial	–2	5	0.3
8	Deep	0	4,5	0.3
	Superficial	–2	6	0.2
9	Deep	–3	6	0.4
	Superficial	–2	4	0.3
10	Deep	1	3	0.3
	Superficial	–2	4,5	0.3
11	Deep	1	4	0.2
	Superficial	–1	3,5	0.2

## Discussion

In this cadaveric study, the feasibility of transferring a motor branch of the PT to the motor branch of the FDP from the UN has been investigated and described. In the event of a failed or impossible high UN repair, other than loss of intrinsic musculature, patients complain of weakness of their hands.^9^, ^10^, ^11^

This is demonstrated by different papers in which demonstrated that UN participated in the contribution of finger flexion, although is not unique nerve required for finger flexion's strength.^12^, ^13^, ^14^ Although numerous nerve transfer techniques have been described in the literature over the past years for the recovery of intrinsic muscle function and sensitive function of the ulnar side of the hand, only one study reported a nerve transfer for UN FDP muscle reinnervation.^7^^,^^15^, ^16^, ^17^, ^18^, ^19^, ^20^, ^21^, ^22^, ^23^, ^24^, ^25^ We believe that motor branches to the pronator teres, commonly used as donors in other nerve transfer techniques, may represent a valid and expendable option as donor to UMBFPD for many reasons.

PT has more than one branch, essential for a nerve transfer procedure. The authors, nonetheless, state that the number of branches to the PTM may not be important because other muscles, such as pronator quadratus, might compensate for loss of function of the PTM.^19^, ^20^, ^21^, ^22^ Our data regarding number, length, diameter, and position of MBPT are in accordance with the findings of several authors.^19^^,^^20^

Moreover, the pronation of the forearm is synergic to finger flexion, leading to an easier and quicker functional recovery. Furthermore, we noted that the longest branch to PT, chosen as the donor in all specimens, was the branch for the superficial belly and arrives in proximity to the target nerve, reducing tension over the suture even in full articular extension of the elbow. In addition, it is important to perform nerve coaptation closer to the target muscle, thus shortening the axonal regeneration distance.

In two of our samples, a common branch for the FCU and FPD was found. These findings regarding UMBFDP length, diameter, and distance from its origin to medial epicondyle are also supported by other anatomical studies.^23^^,^^24^^,^^26^^,^^27^ In this article, the authors found an anatomical configuration similar to ours for half of the specimens, namely, one branch for FPD or a common trunk. For the other half of specimens, in few cases, more than one branch for the FDP was present.

From a technical point of view, when a common branch was found in the presented series, the target branch was dissectable up to the origin of the common trunk; thus, in this article, it was possible to perform the transfer even in such cases presenting the common trunk. As stated above, in few cases, more than one branch was describe. Although we did not encounter situations like this, it is suggested to cautiously explore the UN in order not to miss further branches, even if rare. An interesting practical aspect concerning the anatomy of the UMBFDP consists of the high mobility of FPD muscles and relative motor branches during flexion and extension of the digit. We noted distal migration of the motor branch when the fingers are extended. This indicates the need to perform nerve coaptation with the finger extended. Moreover, the longest donor nerve should be harvested to avoid excessive suture tension or the unnecessary need for nerve graft.

In our study, we did not carry out a histological evaluation with axon counts for either the recipient or donor branch. Instead, we looked at data in the literature. The ulnar branch to the

FPD contains 753 ± 364 myelinated axons; however, 646 (min 326, max 1,224) myelinated axons are noted for PPT and 599 (360–778) for DPT.^18^^,^^20^ These previous data suggest and support the possibility to reinnervate the target muscles adequately.

The present study has several limitations that should be addressed in future anatomical and clinical research. First, this is a feasibility study conducted on specimens that simulate—but do not perfectly replicate—living patients. The laxity of muscles, fascia, and joints in cadaveric specimens may differ from that observed in live surgery, potentially affecting the findings. Therefore, our conclusions regarding the feasibility of nerve transfer should be confirmed in actual surgical settings.

Another limitation is the small sample size. Rare anatomical variations, particularly in the motor branches of the FDP, are well-documented.^23^, ^24^, ^25^ Surgeons should remain aware of these variations to avoid missing motor nerve branches during procedures.

Additionally, we did not examine how nerve transfer outcomes might change following anterior transposition of the UN. It is our view that such a procedure should not affect the location where the nerve coaptation is performed. In fact, when the recipient nerve is dissected to its origin and transected, it is then moved anteriorly over the flexor pronator muscle mass to meet the donor nerve in the same position where the UN is placed after an anterior transposition. Therefore, we believe this approach would not yield considerable improvement. This aspect warrants further investigation.

We believe that transferring one MBPT to the UMBFPD is a viable option, particularly given the short distance and diameter match between the two motor branches, the relative ease of access to both nerves, and the frequent availability of an expendable MBPT donor. This approach could be considered for restoring FDP motor function of the fourth and fifth digits in cases of high UN injuries. It may also represent a valuable solution for tetraplegic patients with an expendable PT, or in cases of brachial plexus injuries involving only the C8–T1 roots.

## Conflicts of Interest

No benefits in any form have been received or will be received related directly to this article.
